# Comparison of UV/H_2_O_2_, UV/PMS, and UV/PDS in Destruction of Different Reactivity Compounds and Formation of Bromate and Chlorate

**DOI:** 10.3389/fchem.2020.581198

**Published:** 2020-09-25

**Authors:** Ying-Hong Guan, Jin Chen, Li-Jun Chen, Xin-Xin Jiang, Qiang Fu

**Affiliations:** School of Water Conservancy and Civil Engineering, Northeast Agricultural University, Harbin, China

**Keywords:** UV/H_2_O_2_, UV/persulfate, UV/peroxymonosulfate, bromate, chlorate

## Abstract

In this study, we compared the decontamination kinetics of various target compounds and the oxidation by-products (bromate and chlorate) of PMS, PDS, and H_2_O_2_ under UV irradiation (UV/PMS, UV/PDS, UV/H_2_O_2_). Probes of different reactivity with hydroxyl and sulfate radicals, such as benzoic acid (BA), nitrobenzene (NB), and trichloromethane (TCM), were selected to compare the decontamination efficiency of the three oxidation systems. Experiments were performed under acidic, neutral, and alkaline pH conditions to obtain a full-scale comparison of UV/peroxides. Furthermore, the decontamination efficiency was also compared in the presence of common radical scavengers in water bodies [bicarbonate, carbonate, and natural organic matter (NOM)]. Finally, the formation of oxidation by-products, bromate, and chlorate, was also monitored in comparison in pure water and tap water. Results showed that UV/H_2_O_2_ showed higher decontamination efficiency than UV/PDS and UV/PMS for BA degradation while UV/H_2_O_2_ and UV/PMS showed better decontamination performance than UV/PDS for NB degradation under acidic and neutral conditions. UV/PMS was the most efficient among the three processes for BA and NB degradation under alkaline conditions, while UV/PDS was the most efficient for TCM degradation under all pH conditions. In pure water, both bromate and chlorate were formed in UV/PDS, small amounts of bromate and rare chlorate were observed in UV/PMS, and no detectable bromate and chlorate were formed in UV/H_2_O_2._ In tap water, no bromate and chlorate were detectable for all three systems.

## Introduction

Sulfate radical (${\text{SO}}_{4}^{\cdot -}$)-based advanced oxidation process has attracted increasing attention as an alternative for traditional hydroxyl radical (HO^·^)-based advanced oxidation process, due to its high oxidation ability (redox potential of 2.5–3.1 V) (Neta et al., 1988) and adjustability to generating HO^·^ via pH manipulation (Guan et al., 2011). ${\text{SO}}_{4}^{\cdot -}$ was generated through activation of peroxymonosulfate (PMS) and peroxodisulfate (PDS) by UV irradiation, electrolysis, base, heat, quinones, ozone, homogeneous, and heterogeneous transition metals (Anipsitakis and Dionysiou, 2004; Furman et al., 2010; Guan et al., 2013; Cong et al., 2015; Zrinyi and Pham, 2017; Chi et al., 2019; Ding et al., 2020; Li et al., 2020; Liu et al., 2020).

PMS, PDS, and hydrogen peroxide (H_2_O_2_) all have similar -O-O- bond and were usually investigated in comparison. A comparison of UV/PDS and UV/H_2_O_2_ was made on decontamination efficiencies. UV/PDS showed a better performance than UV/H_2_O_2_ on the removal of carbamazepine (CBZ), 2,4-bromophenol, ofloxacin (OFX), ibuprofen, and cylindrospermopsin (CYN) (He et al., 2013; Yang et al., 2017; Sun et al., 2019; Luo et al., 2019; Xiao et al., 2020a). In studying the degradation of beta amide antibiotics, TOC removal by UV/PDS was slightly better than that by UV/H_2_O_2_ (He et al., 2014). The better performance of UV/PDS on decontamination than UV/H_2_O_2_ was mainly ascribed to two factors: (1) the higher quantum yield of PDS (Φ = 0.7 mol Einstein^−1^) than that of H_2_O_2_ (Φ = 0.5 mol Einstein^−1^) and (2) lower steady-state concentration of HO^·^ than ${\text{SO}}_{4}^{\cdot -}$ (Yang et al., 2017). The comparison of three UV/peroxide processes (UV/PMS, UV/PDS, and UV/H_2_O_2_) was carried out on destruction of atrazine (ATZ), and it was found that the degradation of UV/PDS on ATZ was more efficient than that of UV/PMS and UV/H_2_O_2_ under the same conditions. It was attributed to the fact that the molar extinction coefficient and quantum efficiency of PDS at 254 nm are higher than those of UV/H_2_O_2_ and UV/PMS (Luo et al., 2015). However, UV/H_2_O_2_ exhibited better performance than UV/PDS on clonidine (CLD) removal that initial degradation rate of CLD was 0.68 and 0.46 μM min^−1^ for UV/H_2_O_2_ and UV/PDS. The removal efficiencies were 86.5 and 78.7% in UV/H_2_O_2_ and UV/PS by the end of experiments, respectively (Xiao et al., 2020b). When removing imidacloprid, UV/PDS showed higher removal efficiency than UV/PMS. This phenomenon was explained by calculating the rate of radical generation and the radical generation rate of UV/PS is higher than that of UV/PMS (Wang Q. F. et al., 2020), while for the removal of tetracycline, degradation efficiency in UV/PMS was higher than that in UV/PDS (Hu et al., 2019). For the three UV/peroxide processes, the superior process varied as target compound changed. The reactivity of target compound would make a sound besides molar extinction coefficient and quantum efficiency. The comparison of UV/peroxide processes need to be performed on target compounds with different reactivity.

When Br^−^- or Cl^−^-containing water was treated by advanced oxidation process, HO^·^ and ${\text{SO}}_{4}^{\cdot -}$ could react with them to form Br^·^ or Cl^·^. Br^·^ or Cl^·^ could react with Br^−^ or Cl^−^ to form ${\text{Br}}_{2}^{\cdot -}$ or ${\text{Cl}}_{2}^{\cdot -}$. HOBr/BrO^−^ or HOCl/ClO^−^ was formed by Br^·^/${\text{Br}}_{2}^{\cdot -}$ or Cl^·^/${\text{Cl}}_{2}^{\cdot -}$ recombination. HOBr/BrO^−^ was reported to be a requisite intermediate in ${\text{BrO}}_{3}^{-}$ formation via pure HO^·^ mechanism (von Gunten and Oliveras, 1998). In UV/PDS, ${\text{BrO}}_{3}^{-}$ was formed significantly in the presence of Br^−^. HOBr/BrO^−^ was also thought as a requisite intermediate in UV/PDS. Br^−^ was initially oxidized by ${\text{SO}}_{4}^{\cdot -}$ to form HOBr/BrO^−^ and the intermediate HOBr/BrO^−^ was then oxidized by ${\text{SO}}_{4}^{\cdot -}$ or photolysis to ${\text{BrO}}_{3}^{-}$ (Fang and Shang, 2012). Addition of organic matters could suppress ${\text{BrO}}_{3}^{-}$ formation by scavenging Br^·^ (Liu et al., 2018; Wang Z. Y. et al., 2020). ${\text{BrO}}_{3}^{-}$ was formed during oxidation of 2,4-bromophenol by UV/PDS while ${\text{BrO}}_{3}^{-}$ was not formed in UV/H_2_O_2_ (Luo et al., 2019). ${\text{BrO}}_{3}^{-}$ was also formed in UV/PMS and a yield of ${\text{BrO}}_{3}^{-}$ reached 100% at PMS concentration of 500 μM (Luo et al., 2020). A substantial conversion of Cl^−^ into ${\text{ClO}}_{3}^{-}$ was observed in UV/PDS at pH 3 and no ${\text{ClO}}_{3}^{-}$ was observed at pH >5. It was proposed that Cl^·^ formed from the reaction between ${\text{SO}}_{4}^{\cdot -}$ and Cl^−^, initiated a cascade of subsequent pH-dependent reactions to form ${\text{ClO}}_{3}^{-}$ (Lutze et al., 2015). In the process of oxidation, ${\text{SO}}_{4}^{\cdot -}$ was the main reaction species, and all chloride chain reactions were initiated by ${\text{SO}}_{4}^{\cdot -}$ (Qian et al., 2016). HOCl/ClO^−^ was observed as an intermediate during the formation of ${\text{ClO}}_{3}^{-}$ in UV/PDS (Hou et al., 2018), while there was no study referring to the formation of ${\text{ClO}}_{3}^{-}$ in UV/PMS process. The comparison of ${\text{BrO}}_{3}^{-}$ and ${\text{ClO}}_{3}^{-}$ formation in the three UV/peroxide processes was also rarely reported.

The objective of this study was to compare (i) decontamination efficiencies of UV/peroxide processes (UV/PMS, UV/PDS, and UV/H_2_O_2_) under various conditions and (ii) the formation of bromate and chlorate experimentally. Benzoic acid (BA), a recalcitrant organic compound, is mainly used in the food and pharmaceutical industries (Rayaroth et al., 2017). It has high reaction rate constants with both HO· and ${\text{SO}}_{4}^{\cdot -}$. Thus, BA was used as a probe to indicate the total oxidation capacity of available HO· and ${\text{SO}}_{4}^{\cdot -}$ (Guan et al., 2011). Nitrobenzene (NB), a refractory pollutant, was used in chemical industry and released into the environment with the amount of about 19 million pounds each year through use, leakage, or industrial accidents (Wei et al., 2019). NB was selected as an indicator for HO^·^ since it has high rate constant with HO· but quite low reaction rate constant with ${\text{SO}}_{4}^{\cdot -}$ (Guan et al., 2011). Trichloromethane (TCM), a kind of disinfection by-product, has low rate constant with HO· and ${\text{SO}}_{4}^{\cdot -}$ (Guan et al., 2018) and was used as representative of low reactivity organic pollutant. Firstly, total oxidation capacity of available HO^·^ and ${\text{SO}}_{4}^{\cdot -}$ in UV/peroxide processes and the inhibition of common radical scavengers [bicarbonate, carbonate, and natural organic matter (NOM)] on the UV/peroxide processes were investigated with BA as probe. Then, decontamination efficiencies were compared among UV/peroxides with BA, NB, and TCM as probes to present the performance of UV/peroxide processes on removal of different reactivity organic pollutants under various typical pH values. Finally, the formation of bromate and chlorate in pure water and tap water was monitored. The results would provide comprehensive comparison of UV/peroxide processes and guideline for selection of advanced oxidation process constructed based on UV disinfection unit.

## Materials and Methods

### Materials

Potassium peroxymonosulfate (PMS), potassium peroxodisulfate (PDS), BA, NB, sodium phosphate dibasic, sodium phosphate monobasic, sodium chloride, and potassium bromide were all ACS reagent grade and purchased from Sigma-Aldrich Company. Bromate standard for IC and chlorate standard for IC were also from Sigma-Aldrich Company. Hydrogen peroxide solution (35% w/w) was purchased from Alfa Aesar. HLPC grade phosphoric acid and methanol are available from DIMA-Tech and Thermo Fisher Science Inc. Gas chromatography (GC) grade chloroform (TCM) was purchased from Tianjin Chemical Reagent Co., Ltd. Suwannee River NOM (1R101N) was obtained from the International Humus Society, and the other reagents were of analytical reagent grade and purchased from China National Pharmaceutical Chemical Reagent Co., Ltd. All solutions were prepared in Milli-Q water (18.2 MΩ cm) produced by Milli-Q Biocel water system.

### Experimental Procedures

All the photochemical experiments were carried out in a cylindrical borosilicate glass container with a low-pressure mercury UV lamp (Heraeus, GPH 135t5l/4, 6 W output, 254 nm). The incident radiation intensities of the UV lamp (*I*_0_) were 1.7 × 10^−6^ Einstein s^−1^ (0.6 L solution) and 1.92 × 10^−6^ Einstein s^−1^ (0.55 L solution). The optical path lengths (*L*) of the two reactor vessels were 2.70 cm (0.6 L solution) and 2.63 cm (0.55 L solution), respectively. Samples were extracted at predetermined intervals for each experiment and quenched with excess ascorbic acid or sodium nitrite. In the cases of tests in tap water, bromide was added to monitor the formation of bromate while no additional chloride was added for monitoring chlorate formation. Water quality parameters of tap water are shown in **Table 5**. All experiments were carried out at room temperature (20 ± 2°C). The error bar represents the standard deviation of repeated experiments.

### Analytical Methods

The concentrations of BA and NB were determined by high-performance liquid chromatography (HPLC) equipped with Waters 2,487 double λ detector and Waters symbol C18 column (4.6 mm × 150 mm, particle size 5 μm). TCM was quantified by gas chromatography (Agilent GC 6890). Details could be found in previous studies (Guan et al., 2011, 2018). Concentrations of anions (chloride, bromide, chlorate, and bromate) were analyzed using a high-pressure ion chromatograph (Dionex Integrion) equipped with a Dionex AS19 column (4 × 250 mm). Isocratic eluent of 20 mM KOH, generated online by Dionex EGC 500 KOH, was used to separate the anions at the rate of 1.0 ml min^−1^ with a suppressor current of 50 mA. The injection volume was 200 μl and detection limits for bromate and chlorate were 0.01 and 0.01 μM. PMS concentration was standardized by iodometric titration (Ball et al., 1967). H_2_O_2_ concentration was standardized based on its absorbance at 240 nm (ε = 40 M^−1^·cm^−1^) (Bader et al., 1988) and PDS concentration was quantified at 254 nm (ε = 20 M^−1^·cm^−1^) (Zhang et al., 2018). Pseudo-first-order rate constant (*k*_0_) of target compound degradation was obtained by fitting data of removal efficiency within 75%.

In some cases, such as UV/PMS and UV/H_2_O_2_ processes at pH 11, the decontamination process could not be well-fitted by first-order reaction kinetics. Herein, we introduced the relative difference of removal efficiency (RDRE) of UV/peroxide processes to depict the inhibition effect of inorganic and organic carbon on UV/peroxide processes, which was calculated based on Equation (1).

(1) $$\begin{array}{l} RDRE=\frac{1}{n}\times \overset{n}{\underset{i=1}{\sum }}\frac{\left(c_{i}/c_{0}-{c_{i}}^{\prime }/{c_{0}}^{\prime }\right)}{1-c_{i}/c_{0}} \end{array}$$

where *i* is the index of sampling times ranging from 1 to *n, n* is the sample number. *c*_*i*_ is the concentration of BA at sample time *i* in the absence of bicarbonate, carbonate, or NOM. *c*_*i*_′ is the concentration of BA at the same sample time *i* in the presence of bicarbonate, carbonate, or NOM. *c*_0_ is the initial concentration of BA in the absence of bicarbonate, carbonate, or NOM, and $c_{0}^{\prime }$ is the initial concentration of BA in the presence of bicarbonate, carbonate, or NOM. Positive values of RDRE indicate the stimulation of additive, and negative values indicate the inhibition of additive. The larger the absolute value is, the stronger the stimulation or inhibition effect is.

## Results and Discussion

### Decontamination in the Presence of Common Radical Scavengers

Inorganic carbon (${\text{HCO}}_{3}^{-}$ and ${\text{CO}}_{3}^{2-}$) and NOM are widely present in surface water and groundwater, and regarded as free radical scavengers, leading to weakening the oxidation of target organic pollutants by advanced oxidation processes (Bennedsen et al., 2012). HO^·^ and ${\text{SO}}_{4}^{\cdot -}$ have different reactivity with inorganic and organic carbon, and the difference of reactivity would lead to a different effect on decontamination efficiency in HO· and ${\text{SO}}_{4}^{\cdot -}$-based oxidation process. BA was used as a probe for both HO· and ${\text{SO}}_{4}^{\cdot -}$ to compare the total oxidation capacity and further investigate the influence of inorganic and organic carbon on total oxidation capacity of the three processes.

**Figure 1:**
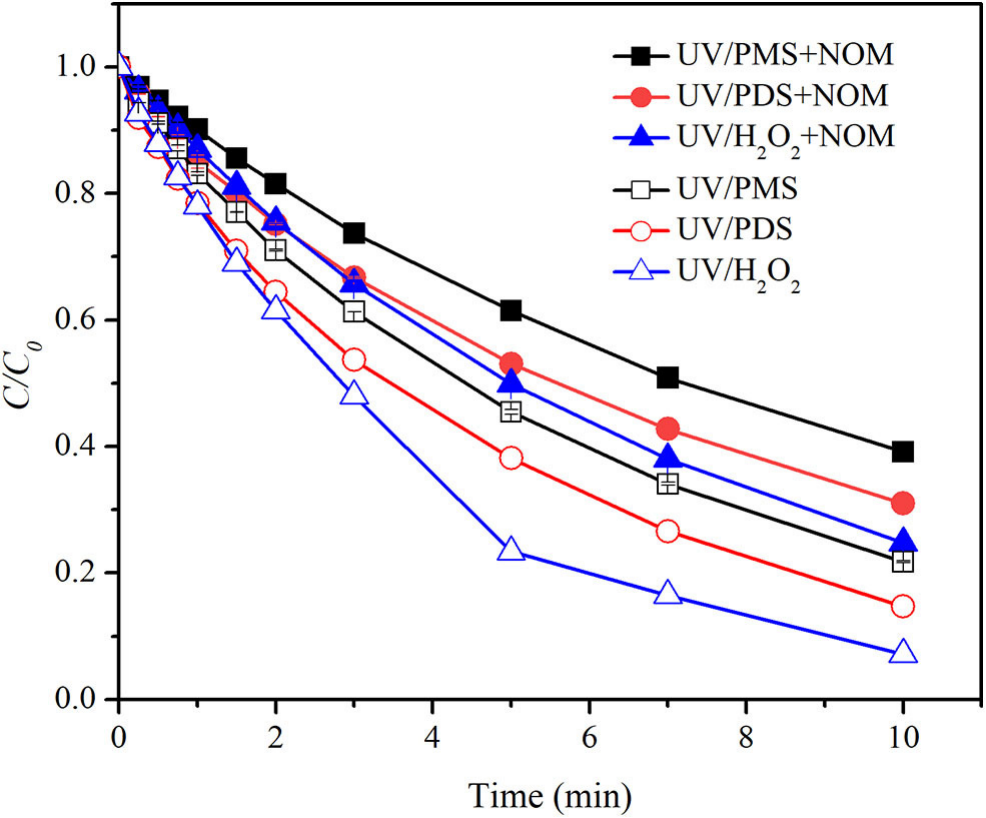
Degradation efficiencies of BA by UV/PMS, UV/PDS, and UV/H_2_O_2_ in the presence of NOM. Conditions: 0.6 L, *I*_0_ = 1.7 × 10^−6^ Einsteins·s^−1^, [PB] = 2 mM, [BA] = 9.0 μM, [PMS] = 100 μM as 1/2 Oxone, [H_2_O_2_] = 100 μM, [PDS] = 100 μM, [NOM] = 2.23 mgTOC·L^−1^, pH = 7.0 ± 0.1, 25°C.

As shown in Figure 1, BA degradation in UV/H_2_O_2_ and UV/PDS was faster than that in UV/PMS under neutral conditions (pH 7). The presence of 2.23 mgTOC·L^−1^ NOM showed inhibition on BA degradation in all the systems. Pseudo-first-order rate constant (*k*_0_) of BA degradation was obtained by fitting data of BA removal within 75% and shown in Table 1. *k*_0_ of BA degradation was 0.00263, 0.00326, and 0.00454 s^−1^ in UV/PMS, UV/PDS, and UV/H_2_O_2_ at pH 7. By adding NOM, values of *k*_0_ were reduced to 0.00160, 0.00203, and 0.00232 s^−1^ in UV/PMS, UV/PDS, and UV/H_2_O_2_, respectively. BA degradation appeared to be still the fastest in UV/H_2_O_2_ after addition of NOM. The presence of NOM led to the relative decrease of *k*_0_ of 39.16, 37.73, and 48.90% in UV/PMS, UV/PDS, and UV/H_2_O_2_, respectively, as shown in Table 2. The values of RDRE between UV/peroxide processes and UV/peroxide processes in the presence of NOM were −0.3468, −0.3069, and −0.3632 in UV/PMS, UV/PDS, and UV/H_2_O_2_ (Table 3). The two indexes, reflecting NOM effect on decontamination, both indicated that NOM showed the largest inhibition on UV/H_2_O_2_ process and the smallest on UV/PDS process. Although HO· has a higher reactivity with BA (5.9 × 10^9^ M^−1^·s^−1^) than ${\text{SO}}_{4}^{\cdot -}$ (1.2 × 10^9^ M^−1^·s^−1^), it also has a higher rate constant with NOM [1.4 × 10^4^ (mgTOC·L^−1^)^−1·^*s*^−1^] than ${\text{SO}}_{4}^{\cdot -}$ [2.2 × 10^3^ (mgTOC·L^−1^)M^−1·^*s*^−1^] (Reactions 2 and 3) (Guan et al., 2018). The value of *k*_radical,BA_/*k*_radical,NOM_ for HO· (ratio of rate constant of HO· and BA to that of HO· and NOM, 4.21 × 10^5^ mgTOC·L^−1·^*M*^−1^) was smaller than that for ${\text{SO}}_{4}^{\cdot -}$ (5.45 × 10^5^ mgTOC·L^−1·^*M*^−1^), which resulted in a slight higher inhibition of NOM on HO^·^ than ${\text{SO}}_{4}^{\cdot -}$. This led to the larger inhibition of NOM on the UV/H_2_O_2_ process and less on the UV/PDS process.

(2) $$\begin{array}{l} \;HO^{\cdot }+NOM\to products\\ \begin{array}{l} k=1.4\times 10^{4}{\left(\text{mgTOC}{\text{L}}^{-1}\right)}^{-1}{\text{s}}^{-1} \end{array} \end{array}$$

(3) $$\begin{array}{l} \;SO_{4}^{\cdot -}+NOM\to products\\ \begin{array}{l} k=2.2\times 10^{3}{\left(\text{mgTOC}{\text{L}}^{-1}\right)}^{-1}{\text{s}}^{-1} \end{array} \end{array}$$

**Figure 2:**
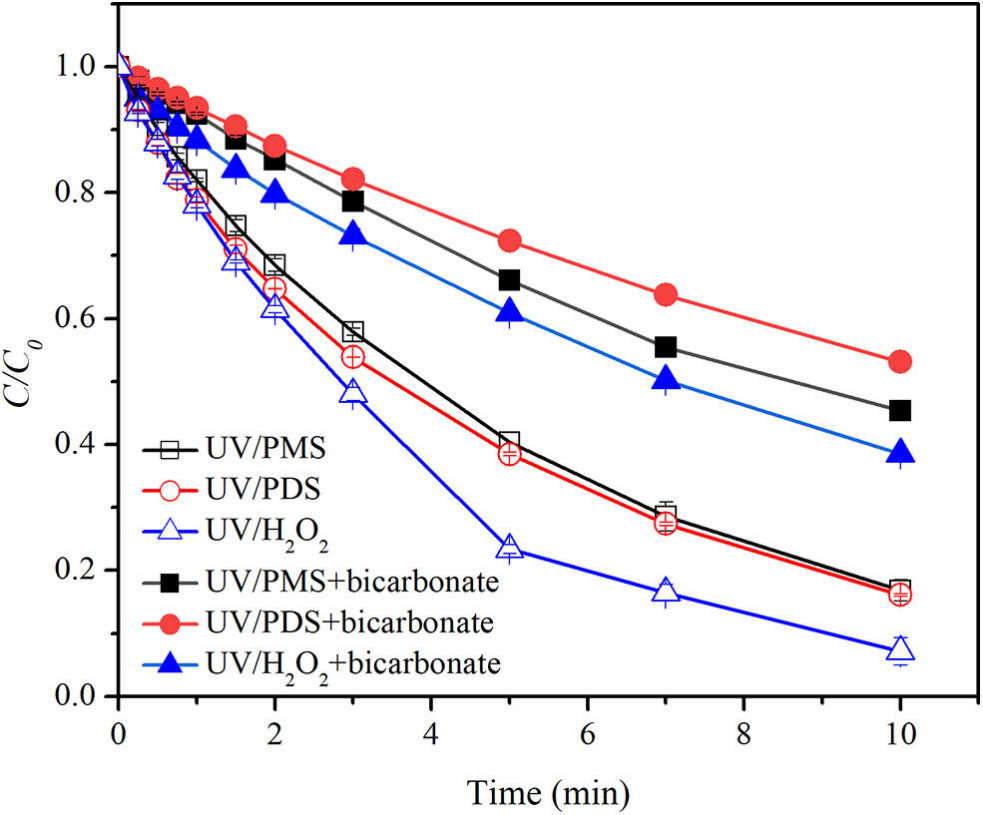
Degradation efficiencies of BA by UV/PMS, UV/PDS, and UV/H_2_O_2_ in the presence of bicarbonate. Conditions: 0.6 L, *I*_0_ = 1.7 × 10^−6^ Einsteins·s^−1^, [PB] = 2 mM, [BA] = 9.0 μM, [PMS] = 100 μM as 1/2 Oxone, [H_2_O_2_] = 100 μM, [PDS] = 100 μM, [${\text{HCO}}_{3}^{-}$] = 10 mM, pH = 8.0 ± 0.1, 25°C.

**Figure 3:**
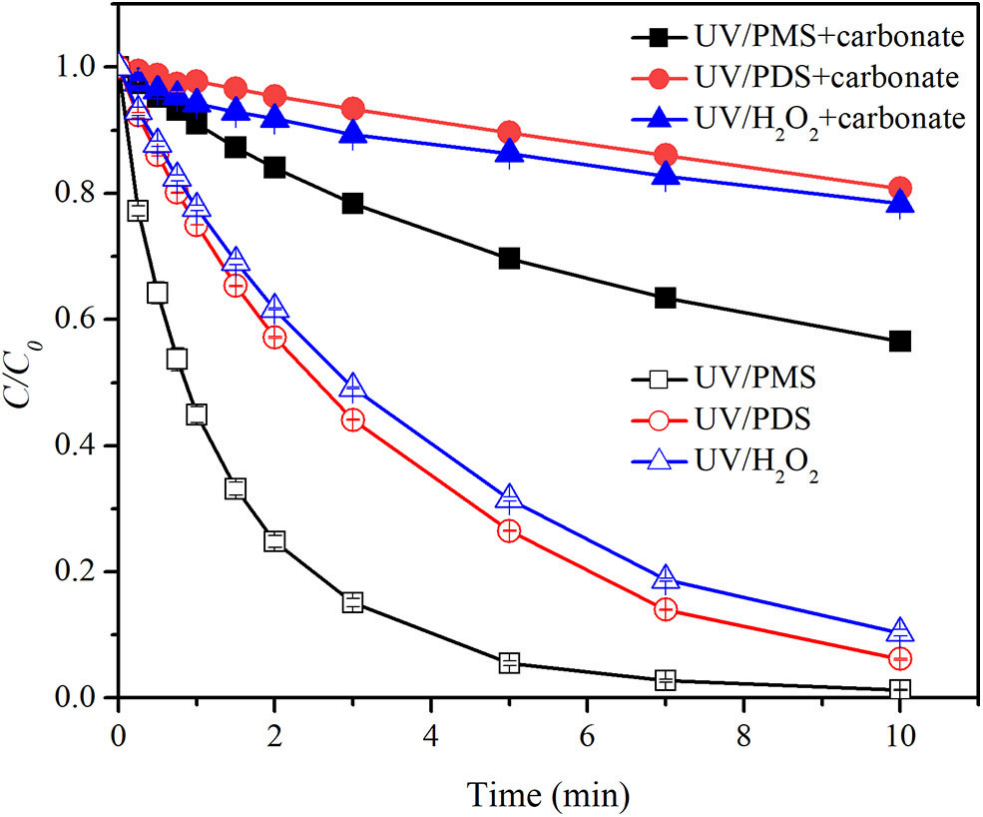
Degradation efficiencies of BA by UV/PMS, UV/PDS, and UV/H_2_O_2_ in the presence of carbonate. Conditions: 0.6 L, *I*_0_ = 1.7 × 10^−6^ Einsteins·s^−1^, [BA] = 9.0 μM, [PMS] = 100 μM as 1/2 Oxone, [H_2_O_2_] = 100 μM, [PDS] = 100 μM, [${\text{CO}}_{3}^{2-}$] = 10 mM, pH = 11.0 ± 0.1, 25°C.

Figure 2 shows the effect of bicarbonate on BA degradation in UV/peroxides. *k*_0_ values of BA degradation were 0.00302, 0.00321, and 0.00409 s^−1^ in UV/PMS, UV/PDS, and UV/H_2_O_2_ at pH 8. *k*_0_ values were decreased to 0.00135, 0.00107, and 0.00164 s^−1^ in UV/PMS, UV/PDS, and UV/H_2_O_2_ by adding bicarbonate (Table 1). UV/H_2_O_2_ was still the most efficient process in the presence of bicarbonate for BA degradation. Figure 3 shows the effect of carbonate on BA degradation in UV/peroxides. *k*_0_ values of BA degradation at pH 11 were 0.01231, 0.00451, and 0.00392 s^−1^ in UV/PMS, UV/PDS, and UV/H_2_O_2_, while *k*_0_ values in the presence of carbonate were 0.00106, 0.00036, and 0.00046 s^−1^ in UV/PMS, UV/PDS, and UV/H_2_O_2_ (Table 1). Carbonate showed significant inhibition on decontamination in all the UV/peroxide processes. In the presence of carbonate, UV/PMS was the most efficient process for BA degradation. This was due to the larger quantity of HO· and ${\text{SO}}_{4}^{\cdot -}$ produced by PMS photolysis since PMS has a quite large molar absorbance coefficient (146.4 M^−1^ cm^−1^) at pH 11 (Guan et al., 2011). Relative decrease of *k*_0_ and RDRE caused by the addition of bicarbonate and carbonate (as shown in Tables 2, 3) indicated that bicarbonate and carbonate showed the largest inhibition on the UV/PDS process. Furthermore, carbonate showed larger inhibition on UV/peroxide processes than bicarbonate, while the difference of bicarbonate inhibition extent among UV/peroxide processes was more obvious than carbonate.

**Table 1:** Pseudo-first-order rate constant (*k*_0_) of BA degradation in the presence and absence of radical scavengers in UV/peroxides obtained by fitting data of BA removal within 75%.

**UV/peroxide** **processes**	**pH 7**		**pH 7, NOM**		**pH 8**		**pH 8, bicarbonate**		**pH 11**		**pH 11, carbonate**	
	***k*_**0**_ (s^**−1**^)**	***R*^**2**^ of**	***k*_**0**_' (s^**−1**^)**	***R*^**2**^ of**	***k*_**0**_ (s^**−1**^)**	***R*^**2**^ of**	***k*_**0**_' (s^**−1**^)**	***R*^**2**^ of**	***k*_**0**_ (s^**−1**^)**	***R*^**2**^ of**	***k*_**0**_' (s^**−1**^)**	***R*^**2**^ of**
		**linear fit**		**linear fit**		**linear fit**		**linear fit**		**linear fit**		**linear fit**
UV/PMS	0.00263	0.99815	0.00160	0.99913	0.00302	0.99785	0.00135	0.99881	0.01231	0.99303	0.00106	0.97997
UV/PDS	0.00326	0.99996	0.00203	0.99629	0.00321	0.99495	0.00107	0.99924	0.00451	0.99917	0.00036	0.99717
UV/H_2_O_2_	0.00454	0.99248	0.00232	0.99996	0.00409	0.99924	0.00164	0.99656	0.00392	0.99911	0.00046	0.95388

**Table 2:** Relative differences of *k*_0_ between UV/peroxide processes and UV/peroxide processes in the presence of additives (radical scavengers) (*k*_0_' – *k*_0_)/*k*_0_.

**UV/peroxide**	**NOM**	**Bicarbonate**	**Carbonate**
**processes**			
UV/PMS	−0.3916	−0.5530	−0.9139
UV/PDS	−0.3773	−0.6667	−0.9202
UV/H_2_O_2_	−0.4890	−0.5990	−0.8827

**Table 3:** Relative differences of removal efficiencies (RDRE) between UV/peroxide processes and UV/peroxide processes in the presence of additives (radical scavengers).

**UV/peroxide**	**NOM**	**Bicarbonate**	**Carbonate**
**processes**			
UV/PMS	−0.3468	−0.5008	−0.7878
UV/PDS	−0.3069	−0.6279	−0.8886
UV/H_2_O_2_	−0.3632	−0.4466	−0.7507

The rate constants of HO^·^ and ${\text{SO}}_{4}^{\cdot -}$ with bicarbonate were 8.5 × 10^6^ M^−1^·s^−1^ and 3.6 × 10^6^ M^−1^·s^−1^ (Reactions 4 and 6) (Zhang et al., 2018). The values of *k*_radical,BA_/*k*_radical,bicarboante_ (ratio of rate constant of radical and BA to that of radical and bicarbonate) for HO^·^ and ${\text{SO}}_{4}^{\cdot -}$ were calculated to be 6.94 × 10^2^ and 3.33 × 10^2^, respectively. As a result, bicarbonate showed a slightly higher inhibition on ${\text{SO}}_{4}^{\cdot -}$ than HO^·^ and correspondingly a larger inhibition on the UV/PDS process. The rate constants of HO^·^ and ${\text{SO}}_{4}^{\cdot -}$ with carbonate were 3.9 × 10^8^ M^−1^·s^−1^ and 6.5 × 10^6^ M^−1^·s^−1^ (Reactions 5 and 7) (Zhang et al., 2018). The values of *k*_radical,BA_/*k*_radical,carboante_ (ratio of rate constant of radical and BA to that of radical and carbonate) for HO^·^ and ${\text{SO}}_{4}^{\cdot -}$ were calculated to be 1.51 × 10^1^ and 1.84 × 10^2^, respectively. As a result, carbonate would show a higher inhibition on HO^·^ than ${\text{SO}}_{4}^{\cdot -}$. Under alkaline conditions (pH 11), majority of ${\text{SO}}_{4}^{\cdot -}$ was converted into HO^·^. Therefore, carbonate exhibited significant inhibition on the three processes and the difference of inhibition extent for UV/peroxides was not as much as bicarbonate.

(4) $$\begin{array}{l} HO^{\cdot }+HCO_{3}^{-}\to CO_{3}^{\cdot -}+H_{2}O\;k=8.5\times 10^{6}{\text{M}}^{-1\cdot }{\text{s}}^{-1} \end{array}$$

(5) $$\begin{array}{l} HO^{\cdot }+CO_{3}^{2-}\to CO_{3}^{\cdot -}+HO^{-}\;k=3.9\times 10^{8}{\text{M}}^{-1\cdot }s^{-1} \end{array}$$

(6) $$\begin{array}{l} SO_{4}^{\cdot -}+HCO_{3}^{-}\to CO_{3}^{\cdot -}+SO_{4}^{2-}+H^{+}\;k=3.6\times 10^{6}{\text{M}}^{-1\cdot }s^{-1} \end{array}$$

(7) $$\begin{array}{l} SO_{4}^{\cdot -}+CO_{3}^{2-}\to CO_{3}^{\cdot -}+SO_{4}^{2-}\;k=6.5\times 10^{6}{\text{M}}^{-1\cdot }s^{-1} \end{array}$$

### Destruction of Different Organic Target Compounds

**Figure 4:**
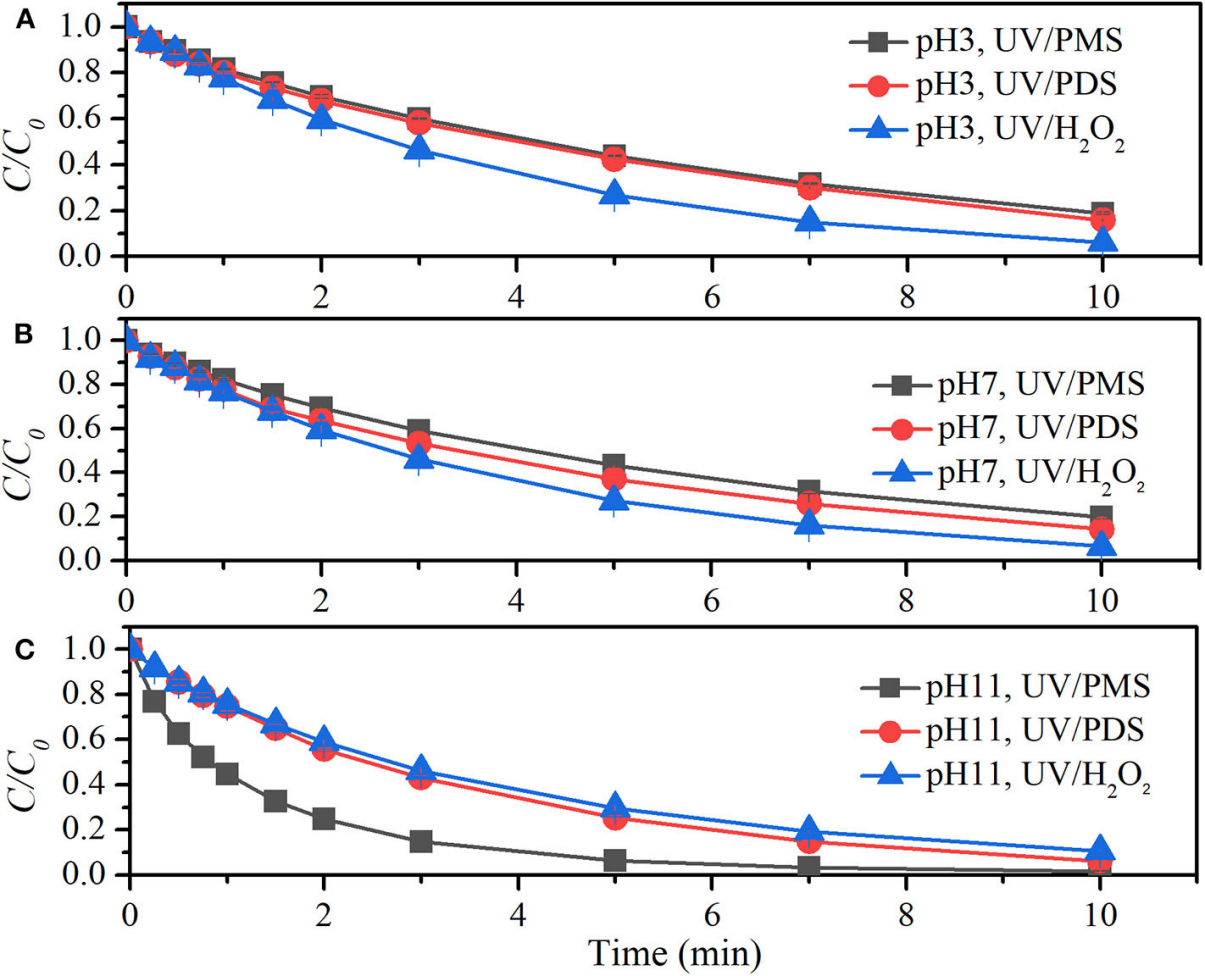
Degradation efficiencies of BA by UV/PMS, UV/PDS, and UV/H_2_O_2_ **(A)** at pH 3, **(B)** at pH 7, and **(C)** at pH 11. Conditions: 0.6 L, *I*_0_ = 1.7 × 10^−6^ Einsteins·s^−1^, [PB] = 2 mM, [BA] = 9.0 μM, [PMS] = 100 μM as 1/2 Oxone, [PDS] = 100 μM, [H_2_O_2_] = 100 μM, 25°C.

Figure 4 shows the comparison of BA degradation by UV/peroxides. *k*_0_ of BA degradation was obtained by fitting data of BA removal within 75% and shown in Table 4. *k*_0_ values of BA degradation by UV/PMS under acidic (pH 3), neutral (pH 7), and alkaline conditions (pH 11) were 0.00279, 0.00281, and 0.01244 s^−1^ at the oxidant concentration of 100 μM. Under the same conditions, the values of *k*_0_ in UV/PDS were 0.00293, 0.00334, and 0.00466 s^−1^, while *k*_0_ values in UV/H_2_O_2_ were 0.00435, 0.00434, and 0.00420 s^−1^ at pH 3, pH 7, and pH 11, respectively. UV/H_2_O_2_ showed higher decontamination efficiencies than UV/PDS and UV/PMS for BA degradation under acidic and neutral conditions, while UV/PMS showed outstanding decontamination efficiency for BA degradation under alkaline conditions. This indicated that UV/H_2_O_2_ has a highest total oxidation capacity of available HO^·^ and ${\text{SO}}_{4}^{\cdot -}$ under acidic and neutral conditions while UV/PMS did under alkaline condition. The results were similar to the reported comparison of clonidine (CLD) removal by UV/H_2_O_2_ and PDS that UV/H_2_O_2_ exhibited better performance than UV/PDS (Xiao et al., 2020b). It was different from the removal of carbamazepine (CBZ), 2,4-bromophenol, ofloxacin (OFX), ibuprofen, and cylindrospermopsin (CYN) by UV/H_2_O_2_ and UV/PDS that UV/PDS showed a better performance than UV/H_2_O_2_ (He et al., 2013; Yang et al., 2017; Luo et al., 2019; Sun et al., 2019; Xiao et al., 2020a). Production rates of radicals from UV/peroxide processes mainly depend on molar extinction coefficients and photolysis quantum yields of peroxides. Molar extinction coefficients of H_2_O_2_ and its dissociated form ${\text{HO}}_{2}^{-}$ were 19.6 and 229 M^−1^·cm^−1^ (Baxendale and Wilson, 1957) while quantum yield of H_2_O_2_ photolysis at 254 nm was Φ = 0.5 (Crittenden et al., 1999). Molar extinction coefficients of ${\text{HSO}}_{5}^{-}$ (monovalent form of PMS) and ${\text{SO}}_{5}^{2-}$ (divalent form of PMS) were 13.8 and 149.5 M^−1^·cm^−1^ while quantum yield of PMS photolysis at 254 nm was Φ = 0.52 (Guan et al., 2011). Molar extinction coefficient and photolysis quantum yield of PDS at 254 nm were reported to be varied in different studies. The values were ε = 20.07 M^−1^·cm^−1^ and Φ = 0.7 (Zhang et al., 2018), ε = 22.07 M^−1^·cm^−1^ and Φ = 0.5 (Qian et al., 2016), and ε = 21.2 M^−1^·cm^−1^ and Φ = 0.567 (Heidt et al., 1948). By considering quantum yield of PDS photolysis as Φ = 0.5, the photo-production rates of total radicals at pH 7 from UV/H_2_O_2_ and UV/PDS were not in big difference, both of which were larger than radical photo-production rate from UV/PMS. This was almost in accordance with the initial degradation of BA at pH 7. With time extension, BA degradation in UV/H_2_O_2_ appeared strengthened as compared with that in UV/PDS. This might be due to the additional production of HO· via reaction between H_2_O_2_ and quinones (Koppenol and Butler, 1985), the intermediate product of BA oxidation. As pH decreased from 7 to 3, degradation rate of BA was almost not affected in UV/H_2_O_2_ and UV/PMS, while degradation rate of BA was slightly slowed down in UV/PDS. As pH increased from 7 to 11, BA degradation was significantly enhanced in UV/PMS. It was mainly due to the increased photo-production of HO· and ${\text{SO}}_{4}^{\cdot -}$, which originated from the increased molar absorption coefficient from 14.3 to 146.4 M^−1^·cm^−1^ (Guan et al., 2011).

**Table 4:** Pseudo-first-order rate constant (*k*_0_) of BA, NB, and TCM degradation in UV/peroxide processes under acidic, neutral, and alkaline conditions.

**Target compounds**	**UV/peroxide processes**	**pH 3**		**pH 7**		**pH 11**	
		***k*_**0**_ (s^**−1**^)**	***R*^**2**^ of linear fit**	***k*_**0**_ (s^**−1**^)**	***R*^**2**^ of linear fit**	***k*_**0**_ (s^**−1**^)**	***R*^**2**^ of linear fit**
BA	UV/PMS	0.00279	0.99765	0.00281	0.99764	0.01244	0.99134
	UV/PDS	0.00293	0.99569	0.00334	0.99510	0.00466	0.99863
	UV/H_2_O_2_	0.00435	0.99912	0.00434	0.99924	0.00420	0.99640
NB	UV/PMS	0.00120	0.98997	0.00106	0.97027	0.00483	0.95544
	UV/PDS	0.00107	0.98811	0.00079	0.97768	0.00204	0.97764
	UV/H_2_O_2_	0.00249	0.99569	0.00204	0.99315	0.00168	0.99028
TCM	UV/PMS	0.00223	0.99713	0.00265	0.99706	0.00117	0.90819
	UV/PDS	0.01524	0.97129	0.01018	0.99438	0.00626	0.99612
	UV/H_2_O_2_	0.00046	0.96875	0.00039	0.94686	0.00033	0.98305

**Figure 5:**
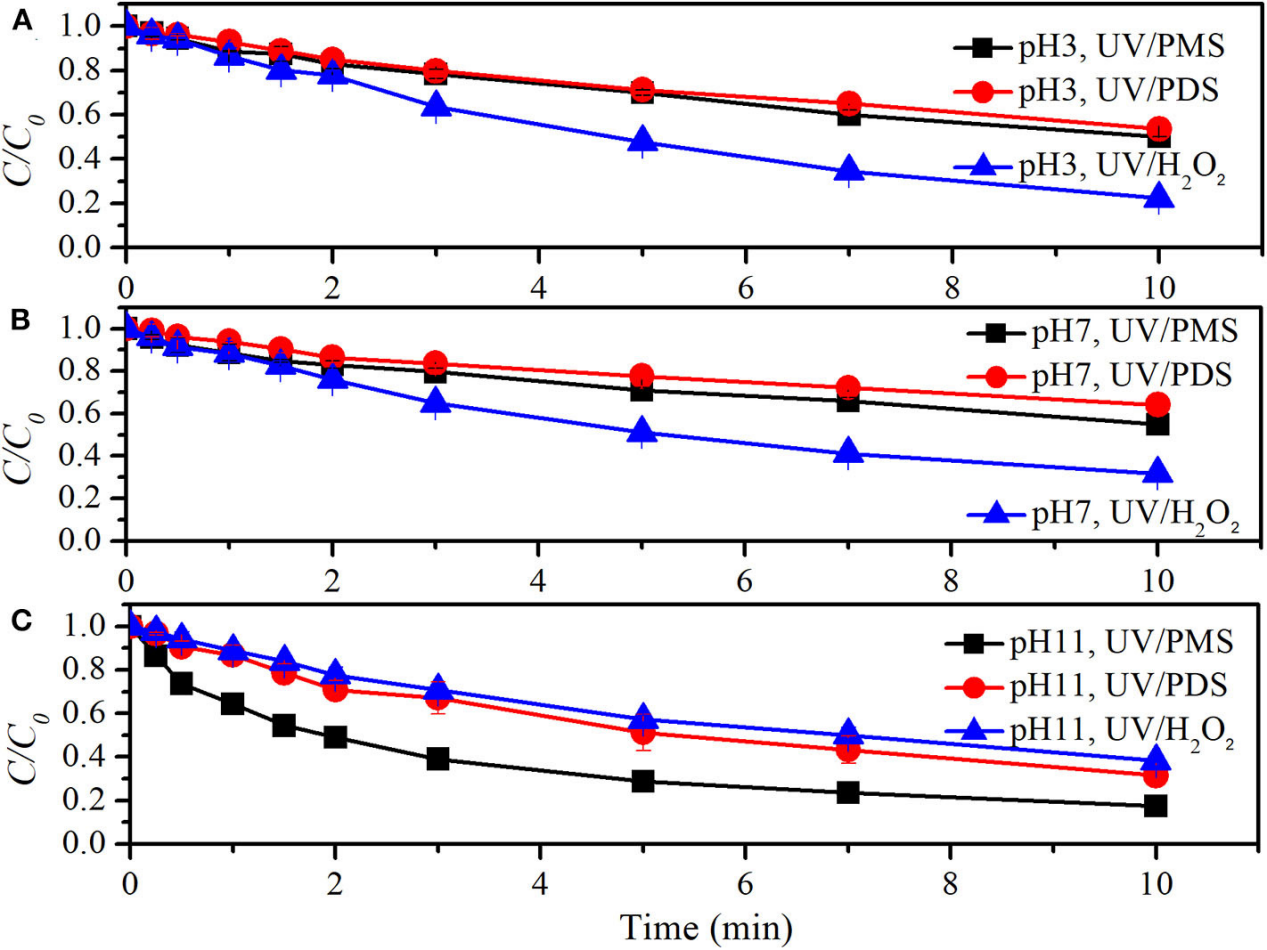
Degradation efficiencies of NB by UV/PMS, UV/PDS, and UV/H_2_O_2_ **(A)** at pH 3, **(B)** at pH 7, and **(C)** at pH 11. Conditions: 0.6 L, *I*_0_ = 1.7 × 10^−6^ Einsteins·s^−1^, [PB] = 2 mM, [NB] = 6.6 μM, [PMS] = 100 μM as 1/2 Oxone, [PDS] = 100 μM, [H_2_O_2_] = 100 μM, 25°C.

Figure 5 and Table 4 show the comparison of UV/peroxide processes on NB degradation. At the oxidant concentration of 100 μM, *k*_0_ values of NB degradation in UV/PMS were 0.00120, 0.00106, and 0.00483 s^−1^ at pH 3, pH 7, and pH 11, respectively. The values of *k*_0_ in UV/PDS were 0.00107, 0.00079, and 0.00204 s^−1^ while *k*_0_ values in UV/H_2_O_2_ were 0.00249, 0.00204, and 0.00168 s^−1^ at pH 3, pH 7, and pH 11, respectively. The performance of UV/peroxides declined in the sequence of UV/H_2_O_2_ > UV/PMS > UV/PDS for NB degradation under acidic and neutral conditions. Meanwhile, UV/PMS also showed excellent decontamination for NB degradation under alkaline conditions. The declined decontamination of UV/H_2_O_2_ from pH 7 to 11 might be due to the increased capture of HO· by hydrogen peroxide and fast depletion of hydrogen peroxide (Crittenden et al., 1999), although the increase of pH would lead to the increased molar absorption coefficient of hydrogen peroxide (Baxendale and Wilson, 1957), intending to increase photo-production of HO·. The rate constants of PDS with ${\text{SO}}_{4}^{\cdot -}$ and HO^·^ were 6.3 × 10^5^ M^−1^·s^−1^ and 1.4 × 10^7^ M^−1^·s^−1^ (Guan et al., 2018). The rate constants of NB with ${\text{SO}}_{4}^{\cdot -}$ and HO^·^ were ≤10^6^ M^−1^ s^−1^ and 3.9 × 10^9^ M^−1^·s^−1^ (Guan et al., 2011). The ratios of *k*_radical,NB_ (rate constant of radical and NB) to *k*_radical,PDS_ (rate constant of radical and PDS) were ≤1.6 and 2.8 × 10^2^ for ${\text{SO}}_{4}^{\cdot -}$ and HO^·^. Correspondingly, the ratios of *k*_radical,NB_·*c*_NB_ to *k*_radical,PDS_·*c*_PDS_ were ≤0.1 and 18.4 for ${\text{SO}}_{4}^{\cdot -}$ and HO^·^ by considering the initial concentrations of NB and PDS. More ${\text{SO}}_{4}^{\cdot -}$ was captured by the parent oxidant PDS than HO^·^. The enhanced decontamination of UV/PDS with pH increasing was mainly due to the conversion of ${\text{SO}}_{4}^{\cdot -}$ into HO·, since HO· has a higher radical usage efficiency than ${\text{SO}}_{4}^{\cdot -}$.

**Figure 6:**
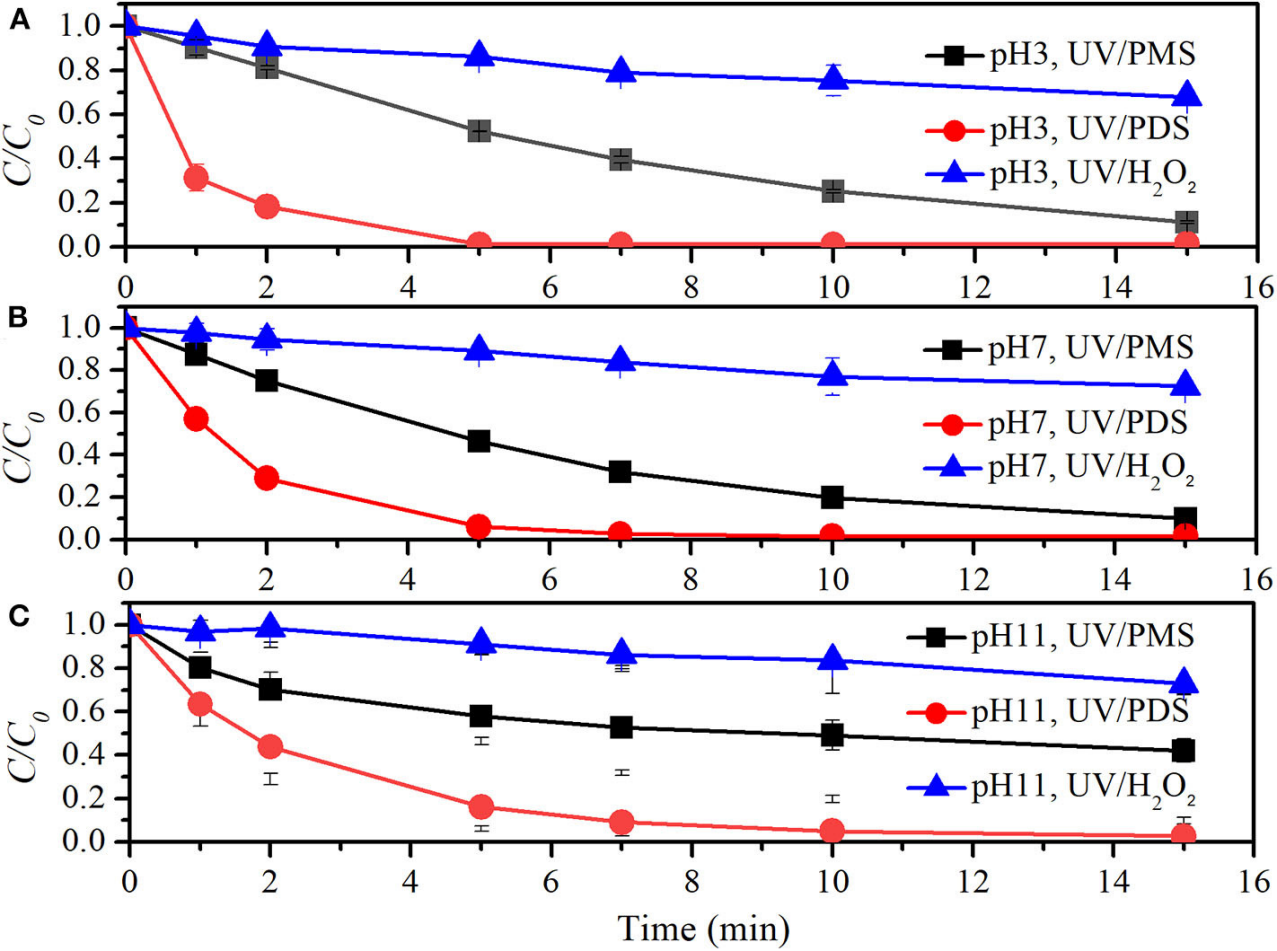
Degradation efficiencies of TCM by UV/PMS, UV/PDS, and UV/H_2_O_2_ **(A)** at pH 3, **(B)** at pH 7, and **(C)** at pH 11. Conditions: 0.6 L, *I*_0_ = 1.7 × 10^−6^ Einsteins·s^−1^, [PB] = 5 mM, [TCM] = 2.1 μM, [PMS] = 0.5 mM as 1/2 Oxone, [PDS] = 0.5 mM, [H_2_O_2_] = 0.5 mM, 25°C.

Figure 6 and Table 4 show the comparison of TCM degradation by UV/peroxides. UV/H_2_O_2_ showed limited degradation of TCM that about 30% of TCM was obtained by 15 min under all the investigated pH values at the oxidant concentration of 500 μM. UV/PDS showed excellent performance on TCM removal that more than 95% of TCM was degraded by 15 min under all pH conditions. *k*_0_ values of TCM degradation at pH 3, pH 7, and pH 11 were 0.00223, 0.00265, and 0.00117 s^−1^ in the UV/PMS process, 0.01524, 0.01018, and 0.00626 s^−1^ in the UV/PDS process, and 0.00046, 0.00039, and 0.00033 s^−1^ in the UV/H_2_O_2_ process, respectively. UV/peroxide performance was in the increased sequence of UV/H_2_O_2_ < UV/PMS < UV/PDS under all pH conditions. During TCM degradation, Cl^−^ was formed as the final product. Cl^·^ would be generated when Cl^−^ coexisted with ${\text{SO}}_{4}^{\cdot -}$ and/or HO^·^ (Lutze et al., 2015; Guan et al., 2018). The limited removal of TCM by UV/H_2_O_2_ might be due to the scavenging of radicals (${\text{SO}}_{4}^{\cdot -}$, HO^·^, Cl^·^, and phosphate radical) by hydrogen peroxide, leading to the low efficiency of radicals for TCM degradation in UV/H_2_O_2_. In UV/PDS, TCM removal decreased as pH increased from 7 to 11. The rate constants of PDS with ${\text{SO}}_{4}^{\cdot -}$, HO^·^, and Cl^·^ were 6.3 × 10^5^ M^−1^·s^−1^, 1.4 × 10^7^ M^−1^·s^−1^, and 8.8 × 10^6^ M^−1^·s^−1^ (Guan et al., 2018). The rate constants of TCM with ${\text{SO}}_{4}^{\cdot -}$, HO^·^, and Cl^·^ were 2 × 10^6^ M^−1^·s^−1^, 6.3 × 10^7^ M^−1^·s^−1^, and 6.6 × 10^7^ M^−1^·s^−1^ (Guan et al., 2018). The ratios of *k*_radical,TCM_ (rate constant of radical and TCM) to *k*_radical,PDS_ (rate constant of radical and PDS) were 3.2, 4.5, and 7.5 for ${\text{SO}}_{4}^{\cdot -}$, HO^·^, and Cl^·^, respectively. The ratios reflected the radical efficiency toward TCM against PDS, similar to the radical participation ratio (RPR) reported in UV/PMS (Guan et al., 2018). In the presence of chloride, ${\text{SO}}_{4}^{\cdot -}$ was fast converted into Cl^·^ and ${\text{Cl}}_{2}^{\cdot -}$ under acidic conditions and increasing pH would lead to conversion of Cl^·^ to HO^·^ (Lutze et al., 2015; Guan et al., 2018). This would lead to the declined TCM degradation as pH increased from 7 to 11. The variation of TCM destruction with pH was similar to the trend of 2-methylisoborneol and geosmin removal vs. pH, which was ascribed to the distribution of phosphate ions (Xie et al., 2015). As pH increased, H_2_${\text{PO}}_{4}^{-}$ dissociated to ${\text{HPO}}_{4}^{2-}$ and ${\text{HPO}}_{4}^{2-}$ has higher rate constants with HO^·^ and ${\text{SO}}_{4}^{\cdot -}$ than H_2_${\text{PO}}_{4}^{-}$. The enhanced scavenging effect of phosphate buffer might also contribute to decrease of TCM removal as pH increased.

The higher removal efficiency for TCM by UV/PDS, as compared with the other two UV/peroxide processes, might be due to the lower scavenging of radicals (${\text{SO}}_{4}^{\cdot -}$, HO^·^, and Cl^·^) by the parent oxidant (PDS) in the reaction system. Based on the analysis of removal of BA, NB, and TCM by the UV/peroxide processes, it could be obtained that the decontamination rate would mainly depend on the molar absorption coefficient and radical quantum yield for the target compounds with high rate constants toward both HO^·^ and ${\text{SO}}_{4}^{\cdot -}$, while the scavenging effect of the parent oxidant for radicals should also be considered besides molar absorption coefficient and radical quantum yield when choosing the superior process of decontamination rate for the target compounds with low rate constants toward ${\text{SO}}_{4}^{\cdot -}$ or toward both HO^·^ and ${\text{SO}}_{4}^{\cdot -}$. It would be suggested that UV/PDS might be a good choice for removing chloro-substituted organic compounds with low rate constant with radicals, while UV/PMS was recommended to be used under alkaline conditions and UV/H_2_O_2_ would be used under acidic and neutral conditions for destructing organic pollutants with high rate constant with radicals.

### Bromate and Chlorate Formation

**Figure 7:**
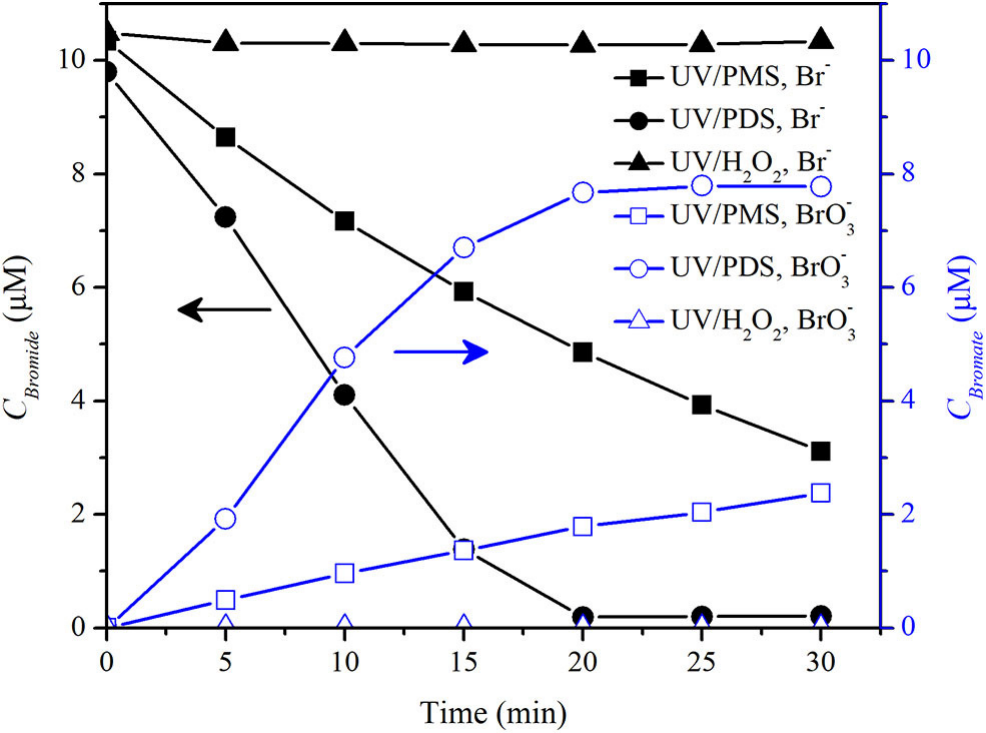
Concentrations of bromide and bromate in UV/PMS, UV/PDS, and UV/H_2_O_2_. Conditions: [PMS] = 200 μM as 1/2 Oxone, [PDS] = 200 μM, [H_2_O_2_] = 200 μM, unbuffered, *I*_0_ = 1.92 μEinstein·s^−1^, *V* = 0.55 L, 20°C.

Bromate and chlorate were reported to be formed in UV/PDS in the presence of bromide and chloride (Fang and Shang, 2012; Lutze et al., 2015). However, whether chlorate was formed in the presence of chloride in UV/PMS system was rarely reported. The comparison of bromate and chlorate formation in UV/peroxide processes was also little reported. Hence, bromate and chlorate formation was comparatively investigated in the UV/peroxide systems in pure water and tap water. Figure 7 shows that ${\text{BrO}}_{3}^{-}$ was obviously formed in UV/PDS in pure water background that ${\text{BrO}}_{3}^{-}$ concentration was about 7.8 μM by 30 min at an initial Br^−^ concentration of 9.8 μM and PDS concentration of 200 μM, while 2.4 μM ${\text{BrO}}_{3}^{-}$ was formed in UV/PMS and no ${\text{BrO}}_{3}^{-}$ was detected in UV/H_2_O_2_. It was consistent with the results that show that no ${\text{BrO}}_{3}^{-}$ was formed during 2,4-bromophenol degradation by UV/H_2_O_2_ (Luo et al., 2019).

In UV/PDS, ${\text{BrO}}_{3}^{-}$ formation was initiated by the reaction between ${\text{SO}}_{4}^{\cdot -}$ and Br^−^ to form Br^·^ and subsequent formation of HOBr/BrO^−^. The intermediate HOBr/BrO^−^ was then oxidized by ${\text{SO}}_{4}^{\cdot -}$ or photolysis to ${\text{BrO}}_{3}^{-}$ (Fang and Shang, 2012). In UV/PMS, Br^·^ and ${\text{Br}}_{2}^{\cdot -}$ formed from Br^−^ by HO^·^ and ${\text{SO}}_{4}^{\cdot -}$ oxidation via Reactions 8 and 9 might react with PMS, since Br^·^/Br^−^ (2.0 V) and ${\text{Br}}_{2}^{\cdot -}$/Br^−^ (1.63 V) have higher redox potential than ${\text{SO}}_{5}^{\cdot -}/HSO_{5}^{-}$ (1.1V) (Eberson, 1982; Neta et al., 1988). The reaction would slow down or hinder the recombination of Br^·^/${\text{Br}}_{2}^{\cdot -}$ to form HOBr/BrO^−^. However, Br^−^ could be oxidized by PMS directly via two electron transfer to HOBr/BrO^−^ with a rate constant of 0.7 M^−1^ s^−1^ via Reactions 10 and 11 (Lente et al., 2009; Zhou et al., 2018). About 2 μM HOBr/BrO^−^ would form by a rough estimation at a PMS concentration of 200 μM and a Br^−^ concentration of 10 μM. The above two aspects might be the reasons for the slow formation of ${\text{BrO}}_{3}^{-}$ in UV/PMS. H_2_O_2_ reacted fast with HOBr/BrO^−^ with a second-order rate constant of 1,900 M^−1^ s^−1^ at pH 6 or 1.5 × 10^4^ M^−1^ s^−1^ (Von Gunten and Oliveras, 1997), which would result in the half-life of HOBr of about 1.8 s and 0.2 s at reaction pH 5.7–6.5 with an initial H_2_O_2_ concentration of 200 μM. It would lead to complete reduction of HOBr to Br^−^. HOBr was a requisite intermediate of bromate (von Gunten and Oliveras, 1998). This resulted in no formation of ${\text{BrO}}_{3}^{-}$ in UV/H_2_O_2_ as shown in Figure 7.

(8) $$\begin{array}{l} SO_{4}^{\cdot -}+Br^{-}\to SO_{4}^{2-}+Br^{\cdot }\;k=3.5\times 10^{9}{\text{ M}}^{-1\cdot }{\text{s}}^{-1} \end{array}$$

(9) $$\begin{array}{l} Br^{-}+HO^{\cdot }\to ...\to Br^{\cdot }\;K\approx 1.1\times 10^{9}{\text{ M}}^{-1\cdot }{\text{s}}^{-1} \end{array}$$

(10) $$\begin{array}{l} Br^{-}+HSO_{5}^{-}\to SO_{4}^{2-}+HOBr\;k=0.7{\text{ M}}^{-1\cdot }{\text{s}}^{-1} \end{array}$$

(11) $$\begin{array}{l} Br^{-}+SO_{5}^{2-}\to SO_{4}^{2-}+HOBr\;k=0.17{\text{ M}}^{-1\cdot }{\text{s}}^{-1} \end{array}$$

(12) $$\begin{array}{l} HOBr\to BrO^{-}+H^{+}\;pKa=8.9 \end{array}$$

**Figure 8:**
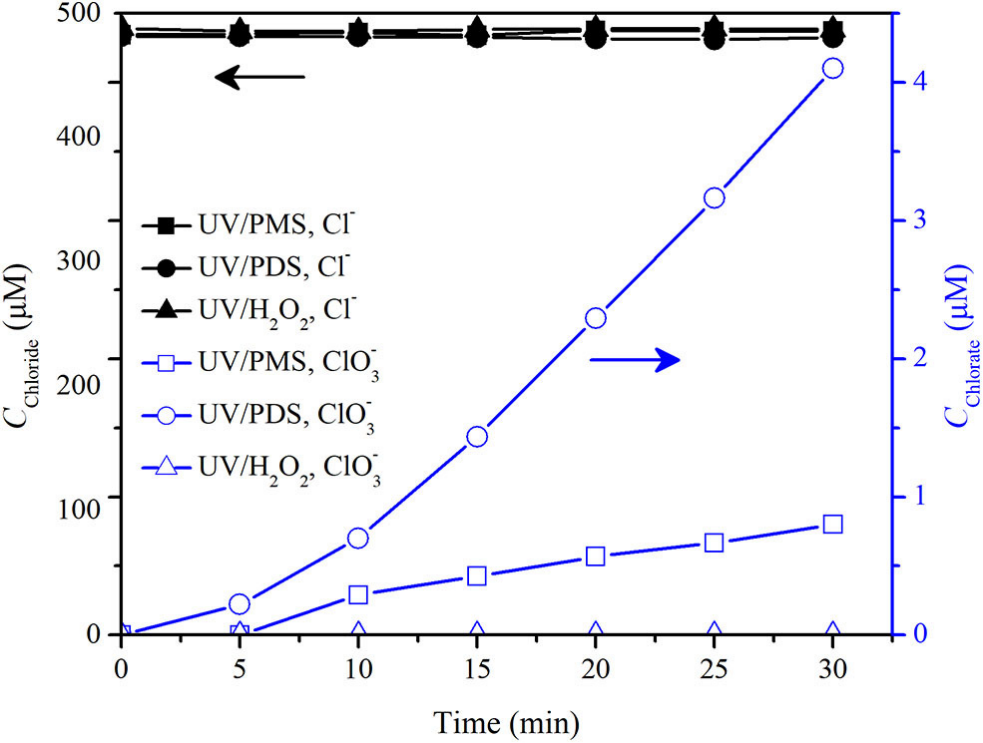
Concentrations of chloride and chlorate in UV/PMS, UV/PDS, and UV/H_2_O_2_. Conditions: [PMS] = 200 μM as 1/2 Oxone, [PDS] = 200 μM, [H_2_O_2_] = 200 μM, unbuffered, *I*_0_ = 1.92 μEinstein·s^−1^, *V* = 0.55 L, 20°C.

Figure 8 shows the ${\text{ClO}}_{3}^{-}$ formation in pure water in the UV/peroxide processes. ${\text{ClO}}_{3}^{-}$ was obviously formed in UV/PDS that 4 μM ${\text{ClO}}_{3}^{-}$ was formed by 30 min in pure water at an initial Cl^−^ concentration of 0.5 mM and a PDS concentration of 200 μM. ${\text{ClO}}_{3}^{-}$ was slightly formed in UV/PMS that 0.8 μM ${\text{ClO}}_{3}^{-}$ was formed by 30 min. No ${\text{ClO}}_{3}^{-}$ was detected in UV/H_2_O_2._

Cl^−^ has a high reaction rate constant with ${\text{SO}}_{4}^{\cdot -}$. It would be quickly oxidized by ${\text{SO}}_{4}^{\cdot -}$ to form Cl^·^ and ${\text{Cl}}_{2}^{\cdot -}$ (Reactions 13 and 14). In UV/PDS, Cl^·^ and ${\text{Cl}}_{2}^{\cdot -}$ recombine with its self or mutually to yield Cl_2_ as Reactions 15–17 (Qian et al., 2016; Guan et al., 2018). Then, Cl_2_ hydrolyzes into HOCl/ClO^−^ via Reactions 18 and 19. HOCl/ClO^−^ is further oxidized by ${\text{SO}}_{4}^{\cdot -}$ and HO^·^ to form ${\text{ClO}}_{3}^{-}$ (Lutze et al., 2015). In the UV/PMS system, ${\text{ClO}}_{3}^{-}$ formation was significantly slow as compared with that in UV/PDS (Figure 8). It might be due to the reaction between Cl^·^/${\text{Cl}}_{2}^{\cdot -}$ and PMS (Guan et al., 2018), which resulted in the reduction of Cl^·^/${\text{Cl}}_{2}^{\cdot -}$ into Cl^−^ by PMS (Reactions 20 and 21) and therefore prevented the formation of Cl_2_ from Cl^·^/${\text{Cl}}_{2}^{\cdot -}$ recombination, cutting down the pathway of ${\text{ClO}}_{3}^{-}$ formation from Cl^·^/${\text{Cl}}_{2}^{\cdot -}$ recombination. However, Cl^−^ could be oxidized by PMS directly via Reactions 22 and 23 although with a low rate constant (Lente et al., 2009; Zhou et al., 2018). This would be the origin of slow chlorate formation in UV/PMS. In UV/H_2_O_2_, Cl^·^/${\text{Cl}}_{2}^{\cdot -}$ might be formed via Reactions 24–26. Meanwhile, Cl^·^/${\text{Cl}}_{2}^{\cdot -}$ reacts fast with H_2_O_2_ and would be reduced readily to Cl^−^ via Reactions 27 and 28 (Guan et al., 2018). Furthermore, HOCl/ClO^−^ could be reduced by H_2_O_2_ (Held et al., 1978) even if it was formed from Cl^·^/${\text{Cl}}_{2}^{\cdot -}$ radical combination, which resulted in the phenomenon that no ${\text{ClO}}_{3}^{-}$ was formed in UV/H_2_O_2_ as shown in Figure 8.

(13) $$\begin{array}{l} SO_{4}^{\cdot -}+Cl^{-}\to SO_{4}^{2-}+Cl^{\cdot }\;k=3.2\times 10^{8}{\text{ M}}^{-1\cdot }{\text{s}}^{-1} \end{array}$$

(14) $$\begin{array}{l} Cl^{\cdot }+Cl^{-}\to Cl_{2}^{\cdot -}\;k=7.8\times 10^{9}{\text{ M}}^{-1\cdot }{\text{s}}^{-1} \end{array}$$

(15) $$\begin{array}{l} Cl^{\cdot }+Cl^{\cdot }\to Cl_{2}\;k=8.8\times 10^{7}{\text{ M}}^{-1\cdot }{\text{s}}^{-1} \end{array}$$

(16) $$\begin{array}{l} Cl_{2}^{\cdot -}+Cl_{2}^{\cdot -}\to Cl_{2}+2Cl^{-}\;k=6.4\times 10^{9}{\text{ M}}^{-1\cdot }{\text{s}}^{-1} \end{array}$$

(17) $$\begin{array}{l} Cl^{\cdot }+Cl_{2}^{\cdot -}\to Cl_{2}+Cl^{-}\;k=2.1\times 10^{9}{\text{ M}}^{-1\cdot }{\text{s}}^{-1} \end{array}$$

(18) $$\begin{array}{l} Cl_{2}+H_{2}O\to HOCl+H^{+}+Cl^{-}\;k\left[{\text{H}}_{2}O\right]=15{\text{s}}^{-1} \end{array}$$

(19) $$\begin{array}{l} HOCl\to ClO^{-}+H^{+}\;pKa=7.6 \end{array}$$

(20) $$\begin{array}{l} Cl^{\cdot }+HSO_{5}^{-}\to SO_{5}^{\cdot -}+Cl^{-}+H^{+}\;k=9\times 10^{8}{\text{ M}}^{-1\cdot }{\text{s}}^{-1} \end{array}$$

(21) $$\begin{array}{l} Cl_{2}^{\cdot -}+HSO_{5}^{-}\to SO_{5}^{\cdot -}+2Cl^{-}+H^{+}\;k=9\times 10^{6}{\text{ M}}^{-1\cdot }{\text{s}}^{-1} \end{array}$$

(22) $$\begin{array}{l} Cl^{-}+HSO_{5}^{-}\to SO_{4}^{2-}+HOCl\;k=2.06\times 10^{-3}{\text{ M}}^{-1\cdot }{\text{s}}^{-1} \end{array}$$

(23) $$\begin{array}{l} Cl^{-}+SO_{5}^{2-}\to SO_{4}^{2-}+HOCl\;k=3.8\times 10^{-4}{\text{ M}}^{-1\cdot }{\text{s}}^{-1} \end{array}$$

(24) $$\begin{array}{l} Cl^{-}+HO^{\cdot }\to ClOH^{\cdot -}\;k_{23}=4.2\times 10^{9} \end{array}$$

(25) $$\begin{array}{l} ClOH^{\cdot -}+H^{+}\to Cl^{\cdot }+H_{2}O\;k_{36}=2.6\times 10^{10} \end{array}$$

(26) $$\begin{array}{l} ClOH^{\cdot -}+Cl^{-}\to Cl_{2}^{\cdot -}+HO^{-}\;k_{38}=1.0\times 10^{5} \end{array}$$

(27) $$\begin{array}{l} Cl^{\cdot }+H_{2}O_{2}\to HO_{2}^{\cdot }+Cl^{-}+H^{+}\;k_{28}=2\times 10^{9} \end{array}$$

(28) $$\begin{array}{l} Cl_{2}^{\cdot -}+H_{2}O_{2}\to HO_{2}^{\cdot }+2Cl^{-}+H^{+}\;k_{33}=6.2\times 10^{6} \end{array}$$

In tap water, no ${\text{BrO}}_{3}^{-}$ or ${\text{ClO}}_{3}^{-}$ was detected with added Br^−^ initial concentration of 10 μM or Cl^−^ initial concentration of 0.5 mM in all three oxidation systems. Water quality parameters are listed in Table 5. NOM in tap water competed for HO· and ${\text{SO}}_{4}^{\cdot -}$ with the rate constants of 1.4 × 10^4^ (mgTOC L^−1^)^−1^ s^−1^ and 2.2 × 10^3^ (mgTOC L^−1^)^−1^ s^−1^ (Guan et al., 2018). Based on the competition kinetics, NOM and bicarbonate would compete for part of HO· and ${\text{SO}}_{4}^{\cdot -}$ with 10 μM Br^−^ and very limited quantity of HO· and ${\text{SO}}_{4}^{\cdot -}$ with 0.5 mM Cl^−^. Br^·^/${\text{Br}}_{2}^{\cdot -}$ and Cl^·^/${\text{Cl}}_{2}^{\cdot -}$ would form. It was reported that Cl^−^ turned ${\text{SO}}_{4}^{\cdot -}$ into HO· at pH 7 in UV/PDS (Lutze et al., 2015) and increasing pH also increased the conversion of ${\text{SO}}_{4}^{\cdot -}$ to HO^·^ and lowered the conversion of ${\text{SO}}_{4}^{\cdot -}$ to Cl^·^/${\text{Cl}}_{2}^{\cdot -}$ in UV/PMS (Guan et al., 2018). The neutral pH condition would not favor the formation of Br^·^/${\text{Br}}_{2}^{\cdot -}$ and Cl^·^/${\text{Cl}}_{2}^{\cdot -}$ and subsequent formation of ${\text{BrO}}_{3}^{-}$ and ${\text{ClO}}_{3}^{-}$ in UV/PDS and UV/PMS in tap water at pH 6.98. Meanwhile, NOM and ${\text{HCO}}_{3}^{-}$ could react with Br^·^/${\text{Br}}_{2}^{\cdot -}$ or Cl^·^/${\text{Cl}}_{2}^{\cdot -}$ and would prevent the formation of HOBr/BrO^−^/Br_2_/${\text{Br}}_{3}^{-}$ or HOCl/ClO^−^/Cl_2_ from Br^−^ and Cl^−^ by radical oxidation, cutting down the formation of ${\text{BrO}}_{3}^{-}$ and ${\text{ClO}}_{3}^{-}$ from the pathway of Br^−^ → Br^·^/${\text{Br}}_{2}^{\cdot -}$ → HOBr/BrO^−^/Br_2_/${\text{Br}}_{3}^{-}$ → → ${\text{BrO}}_{3}^{-}$ and Cl^−^ → Cl^·^/${\text{Cl}}_{2}^{\cdot -}$ → HOCl/ClO^−^/Cl_2_ → → ${\text{ClO}}_{3}^{-}$. Although Br^−^ and Cl^−^ might be oxidized directly to HOBr/BrO^−^/Br_2_/${\text{Br}}_{3}^{-}$ or HOCl/ClO^−^/Cl_2_ via two-electron transfer by PMS (Lente et al., 2009; Zhou et al., 2018), NOM and bicarbonate in tap water would compete for most of ${\text{SO}}_{4}^{\cdot -}$, HO^·^, Br^·^/${\text{Br}}_{2}^{\cdot -}$, or Cl^·^/${\text{Cl}}_{2}^{\cdot -}$ with ${\text{BrO}}_{3}^{-}$/${\text{ClO}}_{3}^{-}$ intermediates formed in low concentration (such as HOBr/BrO^−^ or HOCl/ClO^−^), hindering the formation of ${\text{BrO}}_{3}^{-}$ and ${\text{ClO}}_{3}^{-}$.

**Table 5:** Water quality parameters of tap water.

**DOC**	**Alkalinity**	**Cl^**−**^ (μM)**	**Br^**−**^ (μM)**	**${\text{NO}}_{3}^{-}$ (μM)**	**pH**
**(mg C/L)**	**(as CaCO_**3**_, mg/L)**				
2.78	44.82	535	N.D.	241	6.98

*N.D., not detected*.

## Conclusions

Oxidation of different reactivity compounds and formation of bromate and chlorate were investigated in UV/peroxide processes (UV/PMS, UV/PDS, and UV/H_2_O_2_), as well as inhibition of inorganic and organic carbon on the three processes. NOM showed the largest inhibition on the UV/H_2_O_2_ process and smallest on the UV/PDS process due to the smaller ratio of rate constants of HO^·^ with BA and NOM than ${\text{SO}}_{4}^{\cdot -}$, while ${\text{HCO}}_{3}^{-}$ and ${\text{CO}}_{3}^{2-}$ exhibited largest inhibition on the UV/PDS process. Furthermore, the inhibition of ${\text{CO}}_{3}^{2-}$ was more significant than ${\text{HCO}}_{3}^{-}$ on all three UV/peroxide processes. The difference of inhibition extent of ${\text{CO}}_{3}^{2-}$ on the UV/peroxides was smaller than ${\text{HCO}}_{3}^{-}$. This was ascribed to the conversion of ${\text{SO}}_{4}^{\cdot -}$ to HO^·^ under alkaline conditions (pH 11) and the high rate constant between HO^·^ and ${\text{CO}}_{3}^{2-}$.

UV/H_2_O_2_ showed higher decontamination efficiencies than UV/PDS and UV/PMS for BA degradation under acidic and neutral conditions, while UV/PMS showed outstanding decontamination efficiency for BA degradation under alkaline conditions. The performance of UV/peroxide processes declined in the sequence of UV/H_2_O_2_ > UV/PMS > UV/PDS for NB degradation under acidic and neutral conditions. Meanwhile, UV/PMS also showed excellent decontamination efficiency for NB degradation under alkaline conditions. UV/peroxide performance for TCM degradation was in the increased sequence of UV/H_2_O_2_ < UV/PMS < UV/PDS under all pH conditions. The high removal efficiencies for TCM by UV/PDS, as compared with the other two UV/peroxide processes, might be due to the lower scavenging of radicals (${\text{SO}}_{4}^{\cdot -}$, HO^·^, and Cl^·^) by the parent oxidant (PDS) in the reaction system.

In pure water background, 7.8 μM ${\text{BrO}}_{3}^{-}$ was formed by 30 min at an initial Br^−^ concentration of 9.8 μM and an oxidant concentration of 200 μM in UV/PDS, while 2.4 μM ${\text{BrO}}_{3}^{-}$ was formed in UV/PMS and no ${\text{BrO}}_{3}^{-}$ was detected in UV/H_2_O_2_. 4 μM ${\text{ClO}}_{3}^{-}$ was formed by 30 min in pure water at an initial Cl^−^ concentration of 0.5 mM and an oxidant concentration of 200 μM in UV/PDS. 0.8 μM ${\text{ClO}}_{3}^{-}$ was formed by 30 min in UV/PMS and no ${\text{ClO}}_{3}^{-}$ was detected in UV/H_2_O_2_. In tap water, no ${\text{BrO}}_{3}^{-}$ or ${\text{ClO}}_{3}^{-}$ was detected with added Br^−^ initial concentration of 10 μM or Cl^−^ initial concentration of 0.5 mM in all three oxidation processes.

Based on the comparison of UV/peroxide processes, it was suggested that UV/PMS would be used in alkaline water bodies and UV/H_2_O_2_ would be suitable under acidic and neutral conditions for destructing organic pollutants with high rate constants toward radicals, while UV/PDS might be efficient for removing chloro-substituted organic compounds with low rate constants toward radicals, but need serious concern on controlling the oxidation time to avoid chlorate formation via over-oxidation.

## Data Availability Statement

The raw data supporting the conclusions of this article will be made available by the authors, without undue reservation.

## Author Contributions

Y-HG: responsible for manuscript writing, experiment design, and data analysis. JC: experiment conduction and data analysis. L-JC and X-XJ: data analysis and validation. QF: supervision, validation, and funding acquisition. All authors contributed to the article and approved the submitted version.

## Conflict of Interest

The authors declare that the research was conducted in the absence of any commercial or financial relationships that could be construed as a potential conflict of interest.
